# Sustained‐release of sclerostin single‐chain antibody fragments using poly(lactic‐co‐glycolic acid) microspheres for osteoporotic fracture repair

**DOI:** 10.1002/jbm.a.36704

**Published:** 2019-05-10

**Authors:** Ming Li, Shifei Li, Jianheng Liu, Xiang Cui, Shudong Zhang, Jian Zhou, Xiumei Wang, Qi Yao

**Affiliations:** ^1^ Department of Orthopaedics Chinese PLA General Hospital Beijing China; ^2^ Department of Orthopedics Beijing Shijitan Hospital Affiliated to Capital Medical University, Peking University Ninth School of Clinical Medicine Beijing China; ^3^ State Key Laboratory of New Ceramics and Fine Processing School of Materials Science and Engineering, Tsinghua University Beijing China

**Keywords:** fracture healing, microspheres, osteoporosis, protein release, sclerostin‐scFv

## Abstract

Osteoporotic fracture is one of the most common bone diseases in middle and old age, as the most serious consequence of osteoporosis. Sclerostin single‐chain antibody fragments (SCL‐scFv) have been proven to promote bone formation by binding to scleroprotein, a natural antagonist of the Wnt pathway, but it is difficult to rule alone due to the weak permeability and immunogenicity. Herein, we prepared poly(lactic‐co‐glycolic acid) microspheres as a sustained‐release vehicle to prolong the activity of SCL‐scFv. The morphology of microspheres were uniform and nearly sphere, loading efficiency and encapsulation efficiency of SCL‐scFv microspheres were 6.28 ± 1.04% and 48.37 ± 8.11%, respectively. Approximately 90% of the SCL‐scFvs were released from the microspheres over 28 days with initial burst releasing (38%) in the first 4 days. Sustained‐release of active SCL‐scFv from microspheres promoted bone marrow mesenchymal stem cells osteogenic differentiation in vitro and enhanced fracture healing in ovariectomized rats by improving bone mass and bone formation in the fracture region. All these findings demonstrate that the microspheres are able to simultaneously achieve localized long‐term SCL‐scFv controlled release and effectively promote bone formation, which provides a promising approach for osteoporotic fracture.

## 1 INTRODUCTION

Osteoporotic fractures, also known as brittle fractures, are a common symptom to osteoporosis, and usually caused by low energy violence (Cummings & Melton, 2002). With the increase in the aging population, the incidence of osteoporosis‐related fractures has skyrocketed. It has been estimated that there were more than 8.9 million osteoporosis‐related fractures annually worldwide (Johnell & Kanis, 2006). Approximately 1/3 women and 1/5 men aged over 50‐year‐old experienced osteoporotic fractures (Kanis et al., 2000; Melton 3rd, Atkinson, O'Connor, O'Fallon, & Riggs, 1998; Melton 3rd, Chrischilles, Cooper, Lane, & Riggs, 1992). These fractures are associated with significant disability and mortality, especially in the case of hip fracture, 27.6–40.5% male and 15.8–23.3% female would be died within the first year, nearly 40% patients may lost their function and need a long‐term care (Morin et al., 2011). Furthermore, due to osteoporosis with an imbalance in bone metabolism, the patients were prone to delayed healing or even nonunion (Cheung, Miclau, Chow, Yang, & Alt, 2016), affecting the quality of life of patients seriously.

The pathological basis of osteoporotic fracture is osteoporosis. For the treatment of osteoporotic fracture, in addition to conservative treatment and operation, anti‐osteoporosis is an important criterion to be taken into consideration (Ferrari, 2018). The current clinical therapy for osteoporosis treatment primarily focuses on suppressing bone resorption and stimulating bone formation (Pinkerton, Thomas, & Dalkin, 2013). Suppressing bone resorption could effectively prevent bone loss and increase bone mineral density (BMD; Lyu et al., 2019). However, for patients suffered from osteoporotic fractures, who have taken anti‐bone resorption drugs for many years, were advised to discontinue anti‐bone resorption drugs and choose osteogenic agents by the guideline recommendations (Qaseem, Forciea, McLean, & Denberg, 2017). The possible reason might be that inhibiting bone resorption impair bone remodeling and thus affect bone healing (Fu, Tang, Hao, & Dai, 2013). Osteogenic agents can increase the collagen secreted by osteoblasts, promote bone formation and matrix mineralization, improve bone reconstruction, and effectively promote callus formation in the fracture area (Esbrit & Alcaraz, 2013). Hence, the optimal osteoporotic fracture therapy should optimize the combination of decreasing bone resorption and stimulating bone formation, especially the latter.

Sclerostin is a secreted protein expressed by osteocytes (Poole et al., 2005). As a negative regulator of bone formation (Semenov, Tamai, & He, 2005), it antagonizes Wnt by binding β‐catenin signaling to the receptors lipoprotein receptor‐related protein‐4 (LRP4), LRP5, and/or LRP6 (Leupin et al., 2011; Niehrs, 2006). Through antagonizing Wnt/β‐catenin signaling pathway, sclerostin could promote femoral fracture healing in ovariectomized (OVX) rats (Zhang et al., 2016). Currently, most sclerostin antibodies are monoclonal antibodies with disadvantages of a high molecular mass, weak permeability and a high degree of immunogenicity. Single‐chain antibody fragments (scFvs) are small antibody fragments with low molecular mass and high affinity for target antigens, and induce minimal antigenicity in recipient hosts, thus overcoming the high immunogenicity of monoclonal antibodies. Our previous study has confirmed that subcutaneous injection of 2.5 mg/kg scFvs twice a week for 12 weeks in a rat model of osteoporosis stimulated bone formation and promoted bone healing (Yao et al., 2014). However, scFvs possessed a short half‐life and were quickly metabolized in vivo, making them to be inconvenience and difficult to play completely biological effects on specific areas. In order to simplify and enhance biological effects of scFvs, it is urgent to develop a novel implantable carrier with long‐term controlled release of scFvs to meet the therapeutic period need of fracture healing.

Poly(lactide‐co‐glycolide) (PLGA) is a common component in Food and Drug Administration‐approved biocompatible copolymer with suitable mechanical properties, adjustable degradation time and nontoxicity, and is widely used in bone tissue engineering. A number of studies have employed PLGA as the artificial microsphere delivery system for cells, growth factors, and small molecule durgs (Liao et al., 2016; Rafiei & Haddadi, 2017; van Houdt, Ulrich, Jansen, & van den Beucken, 2018). It has been shown to effectively improve the targeting, stability and biological activity of the loading materials. In particular, the application of composite slow‐release carriers greatly improves the efficiency of loading materials and has unique advantages in the application of bone tissue regeneration. However, the application of PLGA loaded scFvs in bone tissue engineering has rarely been studied.

Hence, in this study, on the basis of successful preparation of active SCL‐scFv (Yao et al., 2014), drug‐loaded PLGA microspheres were prepared using a conventional water‐in‐oil‐in‐water (w/o/w) emulsion solvent evaporation method. Microsphere morphology, loading efficiency, microsphere yield, encapsulation efficiency, and in vitro release behaviors were examined. The microspheres were co‐cultured with rat bone marrow mesenchymal stem cells (BMSCs) and then conducted them to treat OVX rats with femoral fractures. Finally, the proliferation and osteogenic differentiation of BMSCs at the gene and protein level were analyzed, and the effects of the microspheres on fracture healing in OVX rats were evaluated by performing bone formation using micro‐computed tomography (CT) scanning and bone morphology parameters.

## 2 MATERIALS AND METHODS

### 2.1 Materials

Sclerostin was provided by Institute of Microbiology, Chinese Academy of Sciences. Lactide and glycolide were purchased from Oriental Tin Yuet Gene Technology Co., Ltd., Beijing, China. Alpha‐mem medium, 10% fetal bovine serum, trypsin, penicillin (100 U/mL), streptomycin (100 mg/mL), dexamethasone, ascorbic acid, sodium glycerophosphate, alizarin red, and alkaline phosphatase (ALP) dye were purchased from Gibco. Cell counting kit‐8 (CCK‐8) was bought from Dojindo, Kumamoto, Japan. RT cDNA Synthesis Kit was bought from Co Win Biotech, Beijing, China. Ultra SYBR Mixture was bought from Low ROX, Co Win Biotech, Beijing, China. Rabbit anti‐collagen I antibody (1:1,000) and olyclonal goat anti‐rabbit IgG H&L Alexa Fluor 790 (1:10,000) were bought from Abcam, Cambridge, MA. ELISA kit was bought from Fisher Scientific. BMSCs were brought from Cyagen Biosciences Inc. Sprague–Dawley (SD) rats (weight: 250 ± 10 g) were obtained from Beijing Vital River Laboratory Animal Technology Co., Ltd. (Beijing, China). Solvents and other compounds were obtained from Chemical Reagent Co., Ltd., China. All reagents were used as received unless otherwise noted.

### 2.2 Fabrication and characterization of the microspheres

SCL‐scFv microspheres were prepared using a water‐in‐oil‐in‐water (w/o/w) solvent evaporation method, it was used to prepare SCL‐scFv microspheres from free‐acid terminated PLGA in this study. Briefly, 100 mg PLGA was prepared via ring‐opening polymerization of L‐lactide and glycolide (nLA:nGA = 50:50, MW 8000), then it was dissolved in 0.7 mL dichloromethane to form an oil phase. SCL‐scFv (10 mg) was suspended in 1 mL of double‐distilled water with bovine serum albumin (BSA) as a protective agent. The suspension served as the internal aqueous phase for the primary emulsion. The internal aqueous phase was added to the oil phase were homogenized with ultrasonic emulsification performed in an ice bath to form W/O emulsion. Then, The emulsion was vortexed with 10 mL (1% PVA) using a magnetic stirrer at 800 rpm for 4 hr at room temperature for solvent evaporation. The hardened microspheres were collected by centrifugation at 3,000 rpm for 10 min and screened to between 40 and 60 μm. The microspheres were obtained followed by washing three times with double‐distilled water and vacuum freeze‐drying. Finally, they were stored at −80°C for long‐term preservation.

Microsphere morphology was observed by scanning electron microscopy (SEM; Hitachi, S‐450, Japan). Prior to sample analysis, the dried microspheres were vacuum‐coated with gold. SCL‐scFv microspheres (10 mg) were dissolved in 1 L of NaOH (1 mol/L) solution. The mixture was then incubated at 37°C in an incubator‐shaker and shaken for 36 hr continuously at 40 rpm. After centrifugation, the amount of encapsulated protein was determined using an Anti‐Sclerostin ELISA kit, as follows. Wells were coated with 2 μg/mL recombinant sclerostin, blocked with 20 g/L BSA at 37°C and then maintained overnight at 4°C. Supernatant was collected and added to each well. An HRP‐labeled sheep anti‐mouse IgG antibody was added to the wells for 1 hr at 37°C, and binding was observed by adding TMB substrate solution. Plates were read at 450 nm on a microplate reader (TECAN, Switzerland) and all the samples were analyzed simultaneously. Loading efficiency = encapsulated amount of SCL‐scFv/total dry weight of microspheres; encapsulation efficiency = encapsulated amount of SCL‐scFv/total amount of SCL‐scFv used for encapsulation; and microsphere yield = amount of recovered microspheres/amount of polymer and drug initially used.

### 2.3 In vitro SCL‐scFv release

SCL‐scFv microspheres (1 mg) were suspended in 50 mL of phosphate‐buffered saline (PBS) and incubated at 37°C in an incubator‐shaker. At defined time intervals (2 hr, 8 hr, 24 hr, 7 days, 10 days, 14 days, 21 days, and 28 days), the samples were centrifuged at 1,500 rpm for 1 min, and 10 mL of the supernatant was removed and replaced with 10 mL of fresh buffer. The protein content in the supernatant was analyzed using an Anti‐Sclerostin Kit. The cumulative percentage of drug released was calculated. The release experiments were performed in triplicate.

### 2.4 In vitro cell viability evaluation

BMSCs were cultured (37°C, 5% CO_2_, saturated humidity) in growth medium containing alpha Minimum Essential Medium supplemented with 10% fetal bovine serum, penicillin (100 U/mL), and streptomycin (100 mg/mL). Cells at the third passage were used for the following experiments. To examine the effects of SCL‐scFv microspheres on cell proliferation, BMSCs were seeded in 100 μL of growth medium at a density of 1 × 10^3^ cells/well in 96‐well plates and divided into two groups. In the control group, growth medium and blank microspheres were added. In the SCL‐scFv microspheres group, growth medium and SCL‐scFv microspheres (50 ng/mL final drug concentration) were added. At defined time intervals (0, 1, 2, 3, 5, 7, 9, 11, and 14 days), 10 μL of CCK‐8 solution was added to each well, and the cells were incubated for another 1 hr at 37°C. The absorbance was measured at 450 nm with a microplate reader.

### 2.5 Evaluation of osteogenic differentiation

Osteogenic differentiation was evaluated by alizarin red and ALP staining with a modified calcium cobalt method. Rat BMSCs were seeded at 3 × 10^4^ cells/cm^2^ in a 6‐well culture plate and divided into three groups. The control group was added with growth medium and blank microspheres. In the positive control group, growth medium, blank microspheres and osteogenic medium were added. In the experimental group, growth medium, osteogenic medium and SCL‐scFv microspheres (50 ng/mL final drug concentration) were added. After 7 days of differentiation, the cells were fixed with 4% paraformaldehyde for 30 min, stained with ALP for 30 min, and washed three times with PBS before microscopic examination. After 14 days of differentiation, calcium deposits were stained with alizarin red.

Total protein was extracted from the cells after 14 days were divided into three groups (control group, positive control group and experimental group). The cells were lysed on ice for 30 min in lysis buffer supplemented with protease inhibitors, followed by a BCA assay to detect the protein concentration. Protein samples were diluted in loading buffer and denatured for 10 min at 100°C prior to electrophoresis. Proteins were separated on SDS‐PAGE gels and transferred to nitrocellulose membranes. The membranes were blocked for 1 hr at room temperature with skim milk. Membrane strips were incubated with rabbit anti‐collagen I antibody overnight at 4°C. After incubation with polyclonal goat anti‐rabbit IgG H&L Alexa Fluor 790, specific reactions were revealed using the Li‐Cor Odyssey Infrared Imaging System and quantified using Odyssey 3.0 analytical Image Studio software.

Osteocalcin was detected in the cell supernatant using an ELISA kit after 14 days among three groups (control group, positive control group, and experimental group). All procedures were performed according to the manufacturer's protocol. The amount of detected protein was expressed as ng/mL of supernatant.

### 2.6 The gene expression assay

Total RNA was extracted from cells three groups cells as described above after 3, 7, and 14 days using TRIzol reagent according to the manufacturer's instructions. RT‐PCR was performed using a Super RT cDNA Synthesis Kit. Briefly, first‐strand cDNAs were synthesized at 42°C for 45 min in a 20 μL reaction mixture using 2 μg of isolated mRNA. After reverse transcription reactions, amplification and detection were performed on a 7500 HT Fast Real‐Time PCR system using Ultra SYBR Mixture. Expression was normalized to that of beta‐actin. Data were analyzed using the comparison Ct (2–ΔΔCT) method and expressed as the fold change compared with the respective control group sample.

### 2.7 Surgical procedures

Twenty‐three‐month‐old female SD rats were randomly assigned to two groups, the control group underwent ovariectomy and received blank microspheres (*n* = 10) and the experimental group underwent ovariectomy and received SCL‐scFv microspheres (*n* = 10). Osteoporosis model was established by ovariectomy. Rats were anesthetized by intraperitoneal injection of 0.1% pentobarbital solution (45 mg/kg) and underwent a bilateral ovariectomy via dorsal incision. Eight million units of penicillin was given daily for 3 days post‐surgery. After 3 months, femur surgery was performed on the left side of each rat, the middle of femur was cut off with a wire saw and then it was fixed with 1‐mm Kirschner wire. Each rat was individually housed in a cage that allowed free movement. The experimental group was treated with microspheres containing 2.5 mg/kg SCL‐scFv once per month for 3 months. The control group was treated with blank microspheres once per month for 3 months. All microspheres were injected directly into the fracture area. This study was approved by the Local Ethics Committee for Animal Care and Use of Beijing Shijitan Hospital, Capital Medical University, in China.

### 2.8 Evaluation of bone regeneration capability

High‐resolution digital radiography (Faxitron MX‐20; Faxitron X‐ray, IL) was carried out at 12 weeks post operation. Healing of the femoral bone was compared between rats in both groups. To compare the BMDs of the fracture zone between the two groups of rats, the intramedullary Kirschner wire and surrounding soft tissues were first removed. Femoral samples were then scanned with a micro‐CT system (uCT‐40, Scanco Medical, Switzerland). The scanning protocol was set at a maximum resolution of 27 μm and a separation of 21 μm. BMD (mg/cc), trabecular bone volume fraction (BV/TV, %) and trabecular thickness (Tb.Th, mm) were used as parameters of the reconstructed model.

The femoral bones of rats from each group at 12 weeks post operation were used to study the trabecular histomorphology by hematoxylin and eosin (H&E) staining. The bone samples were removed and fixed in 4% neutral‐buffered formalin for 24 hr, followed by a 1‐week decalcification at 4°C using a 10% ethylenediaminetetraacetic acid solution (pH 7.4). After 12 hr, the samples were dehydrated, paraffin‐embedded, and sectioned. The samples were deparaffinized with xylene and dehydrated in a series of increasing concentrations of alcohol before staining with H&E.

### 2.9 Statistical analysis

Experimental data were expressed as the mean ± *SD* and analyzed with SPSS 20.0 (SPSS, IL) software, using the Student's *t* test or one‐way analysis of variance followed by the Bonferroni post‐test when necessary (*p < 0.05, **p < 0.01).

## 3 RESULTS

### 3.1 Characterization of SCL‐scFv microspheres

**Figure 1:**
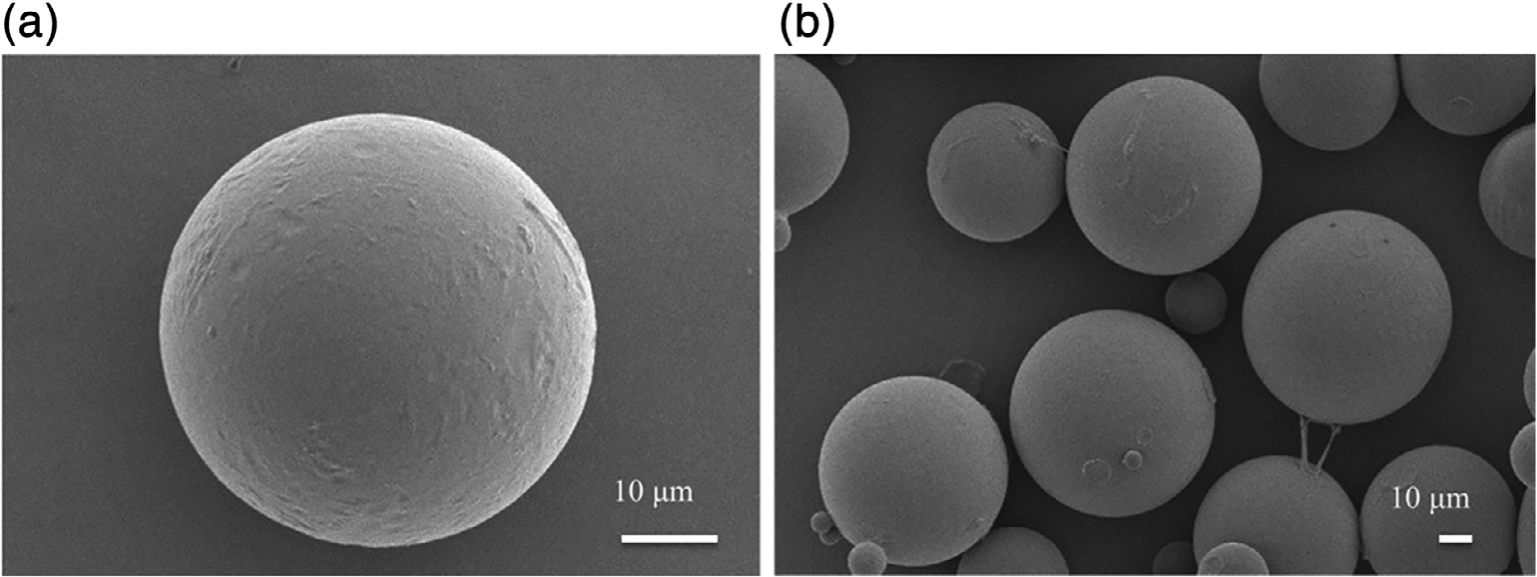
(a and b) SEM images of the microspheres

**Figure 2:**
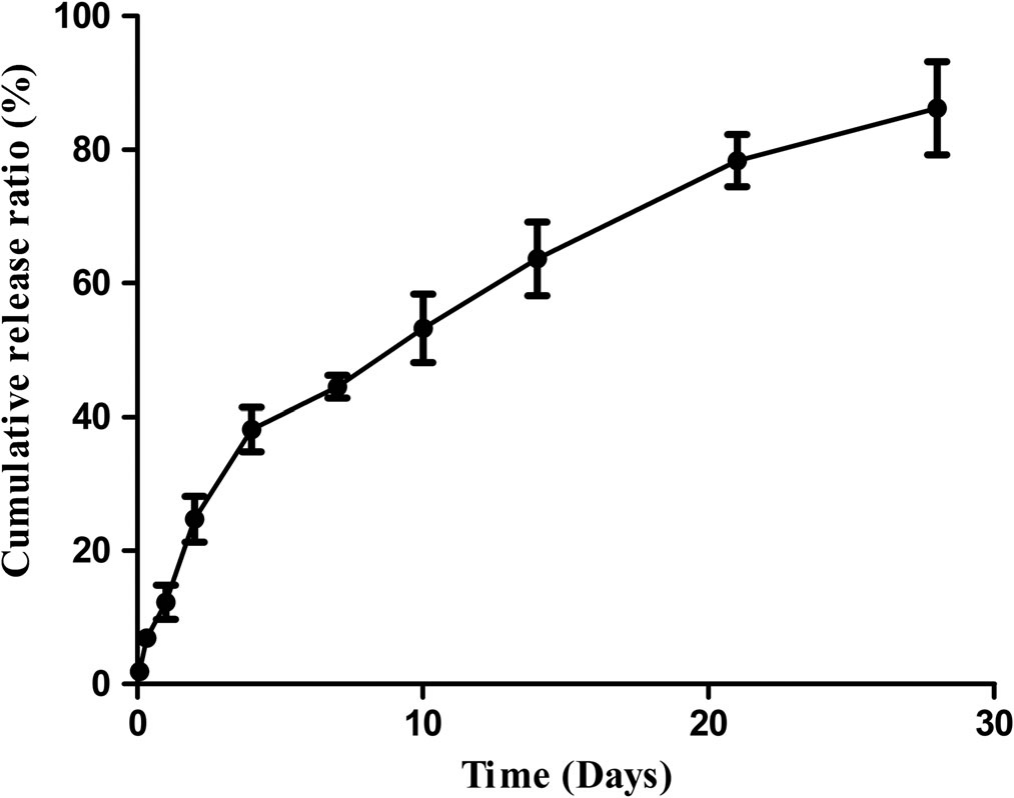
Microsphere release curve

SEM images of the microspheres (Figure 1a,b) showed that they were uniform, nearly circular, and nonadherent. The diameter of microspheres was 51.6 ± 9.8 μm. The microsphere yield, loading efficiency, and encapsulation efficiency of SCL‐scFv microspheres were 70.03 ± 1.3%, 6.28 ± 1.04%, and 48.37 ± 8.11%, respectively. Figure 2 showed the percentage of cumulative SCL‐scFvs released from microspheres at different time points over 28 days. The released SCL‐scFvs in the first 4 days reached approximately 38%, which revealed a characteristic of the burst release. After this initial burst release, the remainders were released with degradation of microspheres. Approximately 90% of the SCL‐scFvs were released from the microspheres over 28 days. These release characteristics could be employed to maintain a local concentration of SCL‐scFv.

### 3.2 Effect of SCL‐scFv microspheres on BMSC proliferation

**Figure 3:**
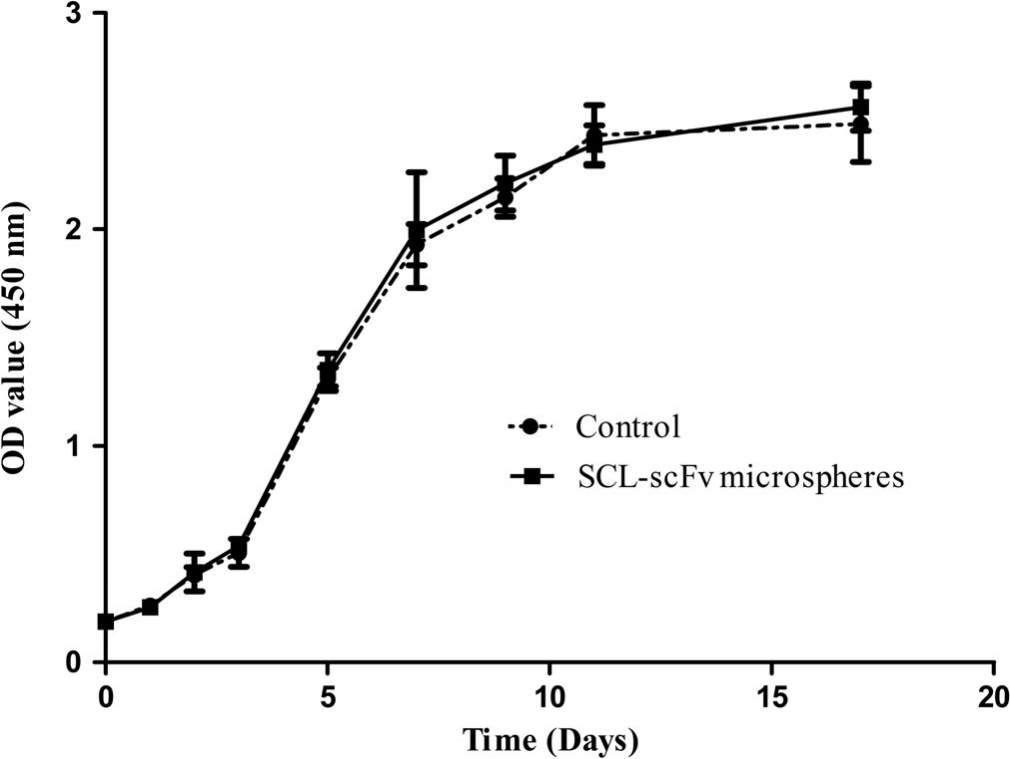
BMSC growth curves. Proliferation was evaluated by a CCK‐8 assay. All experiments were performed at least three times with duplication within each individual experiment

The effect of SCL‐scFv microspheres on cell proliferation was examined by using a CCK‐8 assay. The growth curve (Figure 3) was in the shape of a S. Cells proliferated slow after a lag period over the first 2 days, then the BMSCs entered the log phase on day 3, with a positive slope in the growth curve. The growth curves of both groups indicated that SCL‐scFv microspheres were nontoxic to BMSCs (*p* > 0.05).

### 3.3 In vitro osteogenic induction and differentiation

**Figure 4:**
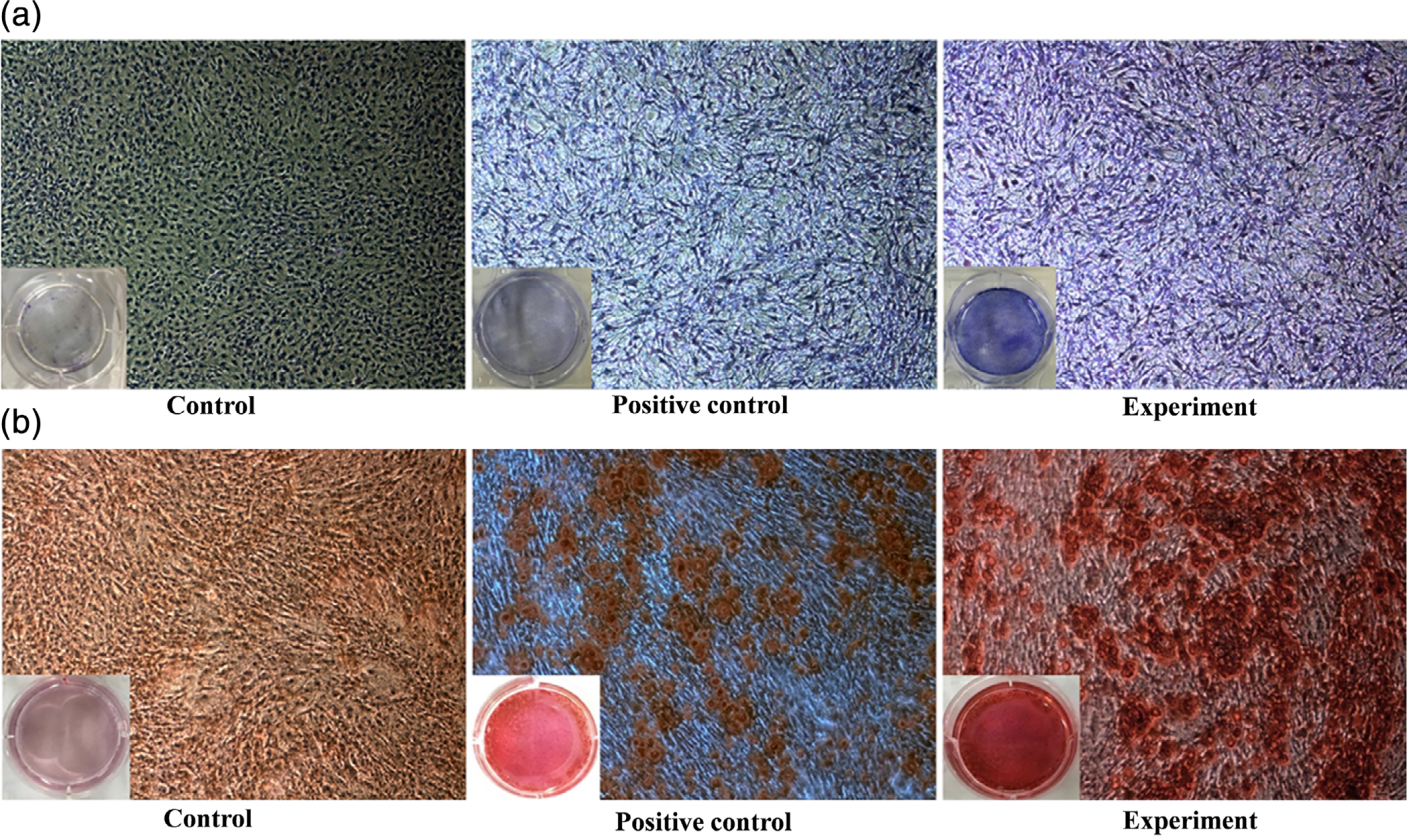
(a) ALP staining of BMSCs in the different groups (×40); (b) Alizarin red staining of BMSCs in the different groups (×40)

Upon ALP staining, cells from the experimental group were mostly dark purple in color (Figure 4a). In the positive control group, the cells were also stained purple, but they were lighter than cells in the experimental group. In the control group, no staining was observed in noninduced cells. Upon alizarin red staining, calcified nodes in the experimental group were dark red (Figure 4b). In the positive control group, the cells were red, but they had fewer calcified nodes than cells in the experimental group. In the control group, no calcified nodes were observed. These results showed that SCL‐scFv microspheres could induce osteogenic differentiation of BMSCs.

### 3.4 Expression of osteogenic‐related genes in BMSCs

**Figure 5:**
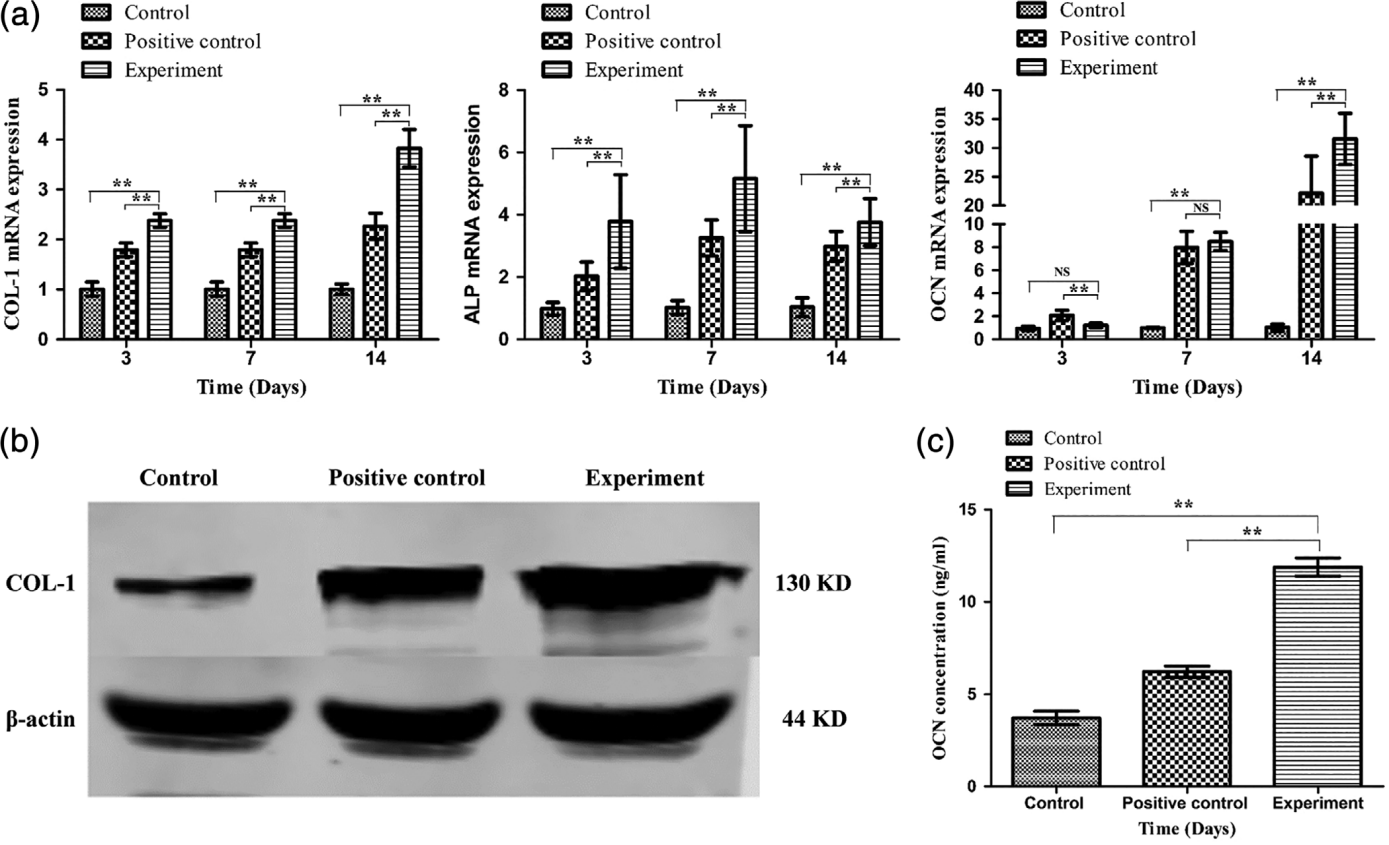
(a) Real‐time PCR detection of osteogenic‐differentiation‐related genes (COL‐I, ALP, OCN) in the different groups of BMSCs. Osteogenic differentiation was induced for 14 days. (b) A western blot assay for evaluating the amounts of COL‐I and β‐actin in the different groups; (c) Expression of OCN in BMSCs in the different groups. Each bar represents the mean of the experiments ± *SD*. *p < 0.05, **p < 0.01. NS, not significant. All experiments were performed at least three times with duplication within each individual experiment

During in vitro BMSC osteogenic differentiation, the expression of several bone‐related genes (COL‐I, ALP, and OCN) was measured by qRT‐PCR (Figure 5a). In the experimental group, expression of the osteogenesis‐related genes COL‐1 increased in a time‐dependent manner, with significant differences observed compared with the positive control group and the control group (*p* < 0.01). The expression levels of OCN after 14 days were significantly higher in the experimental group than in the positive control group and the control group (*p* < 0.05). ALP expression, which is considered a bone regeneration biomarker in the early stages of osteogenesis, was shown to be peaked on day 7 (*p* < 0.01).

### 3.5 Expression of osteogenic‐related proteins in BMSCs

To detect the expression of the osteogenesis‐related proteins COL‐1 and OCN in the three groups, western blot and ELISA experiments were carried out in BMSCs treated with SCL‐scFv microspheres for 14 days. The expression of COL‐I was increased in the experimental group compared with the positive control group and the control group (Figure 5b). The experimental group exhibited significantly increased OCN expression compared with the positive group and the control group (Figure 5c; *p* < 0.01).

### 3.6 Effect of SCL‐scFv microspheres on femur fracture reconstruction

**Figure 6:**
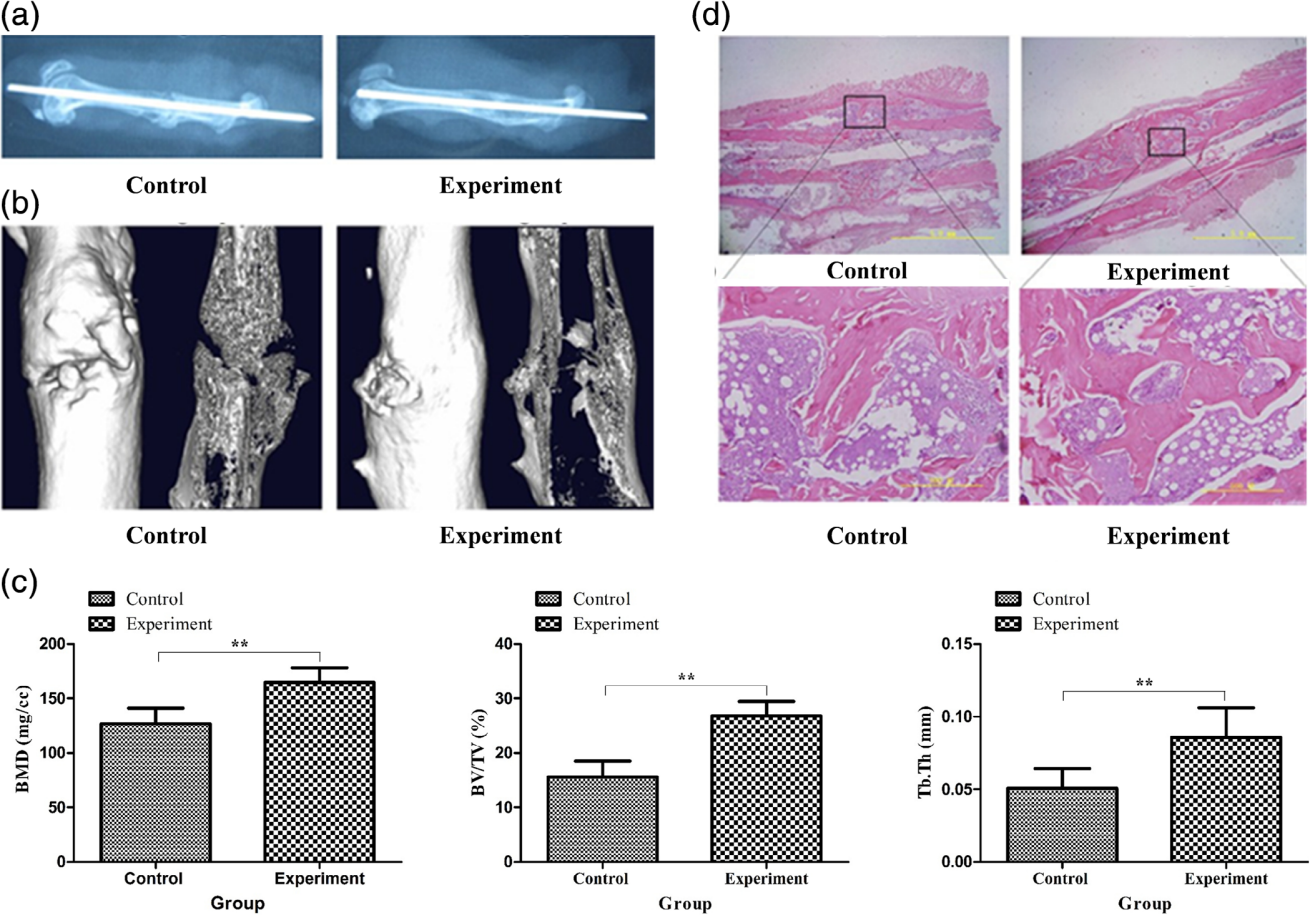
SCL‐scFv microspheres increased bone mass in the fracture region after 12 weeks of treatment. (a) Radiograph of the fracture region at week 12 in the two groups. (b) 3D reconstruction images of the fracture region at week 12 in the two groups. (d) Histological analysis of femoral bone tissue regeneration at week 12 in the two groups. (c) Analysis of BMD, BV/TV, and Tb.Th of the fracture region at 12 weeks in the two groups. Each bar represents the mean ± *SD*. *p < 0.05, **p < 0.01

The fracture regions of femurs from the two groups of rats were examined by radiography and micro‐CT analysis after treatment with SCL‐scFv microspheres for 12 weeks. Compared with the control group, the cortex in the experimental group was continuous, and the fracture line was indistinct (Figure 6a). As shown in Figure 6b, the reconstructed mineralized calluses in the fracture region of the control group were less than that in the experimental group. SCL‐scFv microspheres significantly accelerated trabecular bone formation (Figure 6c), as reflected by increased bone density (*p* < 0.01), BV/TV and Tb.Th were also significantly higher than control group (*p* < 0.01). H&E staining (Figure 6d) showed that the experimental group exhibited more osseous callus and osteoid formation. There were also more bone trabeculae in the experimental group, and the continuity was better than that in the control group. In addition, bone marrow and the marrow cavity had formed in the experimental group. These results indicated that treatment with SCL‐scFv microspheres promoted fracture healing in OVX rats.

## 4 DISCUSSION

Currently, the treatment of osteoporotic fracture is mainly based on anti‐osteoporosis. Sclerostin antagonism stimulates osteogenesis and significantly improves bone metabolism, and does not stimulate osteoclastic activity in the meantime, which has deemed to promoting bone formation (Appelman‐Dijkstra & Papapoulos, 2016; Liu et al., 2016). The treatment of osteoporosis by using anti‐sclerostin antibodies has recently been an important research topic, scFvs have significant advantages due to its high affinity and minimal antigenicity. However, it need to be injected frequently for the short half‐life, which leads to cumbersome use and local drug concentration fluctuation. Hence, sustained‐release of scFvs combined with microspheres have attracted widespread attentions.

In this study, we prepared PLGA microspheres loaded with SCL‐scFv. The manufactured SCL‐scFv microspheres were smooth and uniform because of adding the albumin as stabilizer to reduce the effect of organic solvents on SCL‐scFv (Peng et al., 2016). The loading efficiency and encapsulation efficiency of SCL‐scFv microspheres were higher than the literature value (Asmus et al., 2015), which guaranteed the local release content of SCL‐scFv. Release of SCL‐scFv from the microspheres exhibited two‐phase kinetics, with a rapid initial release phase and a subsequent sustained‐release phase. The burst release was probably related to the excess drug particles released rapidly from matrix into buffer (Li et al., 2011). Approximately 90% of the SCL‐scFvs were released within 28 days, which just match with the period of fracture callus formation. This long‐term release characteristic could be employed to maintain a local concentration of SCL‐scFv to achieve the biological effects.

BMSCs are multipotent cells which originate from the mesenchyme during development and are able to proliferate and differentiate into osteoblasts. BMSCs play a very important role in bone repair and remodeling (Heino & Hentunen, 2008). Therefore, we used SCL‐scFv to block the Wnt/β‐catenin signaling pathway and assessed the expression of osteogenic markers (COL‐І, ALP, OCN and mineralized nodules) in BMSCs during osteogenic differentiation. SCL‐scFv microspheres induced BMSCs osteogenic differentiation, increasing the in vitro expression of the osteogenesis‐related markers ALP, COL‐І and OCN. ALP is a marker of osteoblastic differentiation and matrix synthesis during BMSC differentiation, and its expression peaked on the seventh day and then decreased, which is consistent with the majority of studies (Wang et al., 2013). COL‐І is a major component of organic bone matrices, and OCN is the main noncollagen protein in the bone matrix. The expression of these proteins indicated that SCL‐scFv microspheres promoted BMSC osteogenic differentiation.

To confirm the effect of fracture healing, SCL‐scFv microspheres were implanted in the femoral fracture model of rats. The rats with the treatment of SCL‐scFv microspheres showed dramatically increased BMDs at the fracture site after 12 weeks. In addition, BV/TV and Tb.Th were higher in the experimental group than in the control group, suggesting that sclerostin neutralization significantly mitigated bone mass loss, which coincide with previous studies (Liu et al., 2016). Compared with our previous study of regular subcutaneous injection scFvs (Yao et al., 2014), the treatment with SCL‐scFv microspheres not only simplified the procedure, but also significantly increased BMD and promoted bone formation, achieving the same effect of promoting fracture healing. H&E staining images of the primary callus further confirmed the repair effect.

However, there was also a limitation to our study. The release characteristics of microspheres in vivo were not evaluated, because it was difficult to quantify the SCL‐scFv concentration using the custom methods. But we evaluated the release characteristics of microspheres in vitro by currently accepted methods, which could be used as an effective approach. New methods will be found to further verify the release in vivo release of SCL‐scFv in the following studies.

## 5 CONCLUSIONS

In this study, we successfully prepared PLGA microspheres loaded with SCL‐scFv, examined their characterization, and tested the effects on BMSC differentiation and fracture healing in OVX rats. The results demonstrated that microspheres had good loading performance and were able to release SCL‐scFv continuously over a long period. The SCL‐scFv microspheres could effectively enhance BMSC proliferation, differentiation and mineralization, as well as increase the expression of bone‐related genes and proteins in vitro. Meanwhile, it promoted fracture healing in OVX rats by improving bone mass and bone reconstruction in the fracture region. Overall, these results supported further investigation in SCL‐scFv microsphere as a potential therapy for osteoporotic fractures.
